# Troxerutin alleviates kidney injury in rats via PI3K/AKT pathway by enhancing MAP4 expression

**DOI:** 10.29219/fnr.v66.8469

**Published:** 2022-05-24

**Authors:** Tongxu Guan, Yingce Zheng, Shengzi Jin, Shuang Wang, Mengxin Hu, Xingyao Liu, Siqi Huang, Yun Liu

**Affiliations:** 1 College of Veterinary Medicine, Northeast Agricultural University, Harbin, P. R. China; 2 College of Life Science, Northeast Agricultural University, Harbin, P. R. China; 3 Heilongjiang Key Laboratory for Laboratory Animals and Comparative Medicine, Northeast Agricultural University, Harbin, P. R. China

**Keywords:** troxerutin, kidney injury, cellular apoptosis, MAP4, PI3K/AKT pathway

## Abstract

**Background:**

Troxerutin is a flavonoid compound and possesses potential anti-cancer, antioxidant, and anti-inflammatory activities. Besides, cisplatin is one of the most widely used therapeutic agents, but the clinical uses of cisplatin are often associated with multiple side effects, among which nephrotoxicity is more common.

**Objective and design:**

This study explored the protective effects of troxerutin (150 mg kg^−1^ day^−1^ for 14 days) against cisplatin-induced kidney injury and the potential mechanism using Wistar rats as an experimental mammalian model.

**Results:**

We discovered that troxerutin could significantly alleviate cisplatin-induced renal dysfunction, such as increased levels of blood urea nitrogen and creatinine (*P* < 0.01), as well as improved abnormal renal tissue microstructure and ultrastructure. Additionally, troxerutin significantly decreased malondialdehyde (MDA), hydrogen peroxide (H_2_O_2_), NO, inducible nitric oxide synthase (iNOS) levels (*P <* 0.01), p-NF-κB p65/NF-κB p65, TNF-α, Pro-IL-1β, IL-6, B cell lymphoma-2 (Bcl-2)/Bcl-xl associated death promoter (Bad), Cytochrome C (Cyt C), Cleaved-caspase 9, Cleaved-caspase 3, and Cleaved-caspase 8 protein levels (*P <* 0.01) in the kidney tissues of cisplatin-treated rats; and increased superoxide dismutase (SOD), catalase (CAT), glutathione (GSH), total antioxidant capacity (T-AOC) activities (*P <* 0.01), IL-10, Bcl-2 protein levels (*P <* 0.01).

**Conclusion:**

These results suggested that the underlying mechanism might be attributed to the regulation of Phosphoinositide 3 kinase/Protein kinase B (PI3K/AKT) pathway via enhancing MAP4 expression to attenuate cellular apoptosis, alleviating oxidative stress and inflammatory response.

## Popular scientific summary

- Troxerutin, found in cereals, coffee, tea, a variety of fruits and vegetables, can alleviate cisplatin-induced renal dysfunction in rats.
- Troxerutin regulate PI3K/AKT pathway via enhancing MAP4 expression to attenuate cellular apoptosis, alleviating oxidative stress and inflammatory response.

Cisplatin, namely cis-diamminedichloroplatinum (II), has been widely used in clinical treatments owing to its advantages such as broad-spectrum anti-cancer activity, effective to hypoxic cells and high efficacy (1). Cisplatin remains one of the most effective chemotherapeutic agents with the advancement in medical technology, despite the emergence of several new therapies for cancer. Currently, it is frequently used in the treatment of ovarian cancer, testicular cancer, uterine cancer, breast cancer, bladder cancer, head and neck cancer, lung cancer, prostate cancer, and brain cancer, etc. (2, 3). In addition, cisplatin is a non-specific classical anti-cancer drug that can act on any cell cycle, and can enhance the effect of other chemotherapeutic drugs used in combination therapy (4). It is worth noting that although cisplatin plays a significant role in the clinical treatment of cancer, long-term administration of cisplatin can lead to the accumulation of a large amount of metal platinum ions in the kidney, leading to toxicity, and the concentration of cisplatin in proximal renal tubular epithelial cells is about five times higher compared to serum (5). Subsequently, renal tubular epithelial cells are damaged, which results in reabsorption dysfunction or decreased glomerular filtration rate, affecting the excretion function of the kidney. A previous study demonstrated that the incidence of renal function deterioration was 25~35%, and a decrease of 20~40% in glomerular filtration rate can be observed in patients treated with a single dose of cisplatin once in a day for 10 days (6). The nephrotoxicity of cisplatin is dose-dependent (7), which limits the possibility of increasing the dose of cisplatin and shortening the treatment period.

Troxerutin is a flavonoid compound with a variety of biological activities, which is copiously found in cereals, coffee, tea, a variety of fruits, and vegetables (8). The molecular structure of troxerutin is complex, and the molecular formula is C_33_H_42_O_19_. Different from other flavonoids, troxerutin is highly soluble in water. Therefore, it not only has free radical scavenging ability, but also can be absorbed easily by the intestinal system with good bioavailability (9). At the same time, its food origin and low toxicity make it a potential functional ingredient in new resource foods. Several preclinical studies demonstrated the protective role of troxerutin in D-galactose (D-gal) (10), 2,2’,4,4’-tetrabromodiphenyl ether (BDE-47) (11), gentamycin-induced (8) nephrotoxicity, BDE-47-induced hepatocyte apoptosis (12), cerebral ischemia/reperfusion (I/R) injury (13), and osteoarthritis (14). Furthermore, troxerutin is endowed with anti-oxidative, anti-inflammatory, and anti-apoptotic activities, and its benefits in the prevention of cancer, diabetes, and neurodegenerative and cardiovascular diseases have been widely noted (15). Dehnamaki et al. (16) found that cisplatin can increase blood urea nitrogen (BUN), Cre, and malondialdehyde (MDA) levels and decrease superoxide dismutase (SOD) and GPx activity. Treatment with troxerutin attenuated these changes, having protective effect against cisplatin-induced nephrotoxicity. However, the protective mechanism of troxerutin on cisplatin-induced renal injury is still unclear. Therefore, the objectives of the present study were to investigate the anti-oxidative, anti-inflammatory, and anti-apoptotic effects of troxerutin on cisplatin-induced kidney injury, and its underlying mechanisms.

## Materials and methods

### Animals

Wistar rats (male) weighing 280 ± 15 g (45-day-old on average) were provided by Liaoning Experimental Animal Resource Center, Shenyang, Liaoning, China. All animals were housed in groups of five in polypropylene cages at Northeast Agricultural University under controlled conditions of temperature (23 ± 1°C), humidity (55 ± 5°C), light/dark cycles (12 h/12 h), well ventilated, free drinking and feeding, and regular replacement of bedding. The rats were fed and adapted for 1 week before the start of experiment.

### Chemicals and kits

Cisplatin was purchased from Sigma Chemical Co. (St Louis, MO, USA). Troxerutin was purchased from Aladdin Biochemical Technology Co., Ltd., analytical standard, 98% (Shanghai, China). The detection kits were purchased from Nanjing Jiancheng Bioengineering Institute (Nanjing, China). The primary and secondary antibodies were purchased from Wanlei Biotechnology Co., Ltd. (Shenyang, China).

### Experimental design and treatment protocol

Forty male Wistar rats were randomly distributed into four groups (10 rats per group). Group 1 (Normal control group, N Ctrl): rats in this group were treated with saline (vehicle) by gastric gavage daily for 14 days, and on the 12th day, they were intraperitoneally injected with saline (vehicle). Group 2 (Troxerutin control group, Tr Ctrl): rats in this group were treated with troxerutin (150 mg kg^−1^ day^−1^) by gastric gavage daily for 14 days, and on the 12th day, they were treated with saline (vehicle) by intraperitoneal injection. Group 3 (Cisplatin and troxerutin-treated group, Cis+Tr): rats in this group were treated with troxerutin (150 mg kg^−1^ day^−1^) by gastric gavage daily for 14 days, and on the 12th day, they were intraperitoneally injected with cisplatin (10 mg kg^−1^ day^−1^). Group 4 (Cisplatin-treated group, Cis): rats in this group were treated with saline (vehicle) by gastric gavage daily for 14 days, and on the 12th day, they were treated with cisplatin (10 mg kg^−1^ day^−1^) by intraperitoneal injection. The doses of troxerutin and cisplatin were based on preliminary experiments (Supplementary Fig. 1).

### Sample collection and preparation

Seventy-two hours after administration of cisplatin, rats from all groups were euthanized (via exsanguinations) under sodium pentobarbital (17) for collection of blood samples (via cardiac puncture) and kidney tissues. The obtained blood samples were placed at room temperature for 30 min and then centrifuged at 3,500 rpm for 10 min at 4°C. The supernatants were used for the determination of BUN and creatinine (Cre) in the serum. The collected kidney tissues were quickly rinsed in ice cold saline and the surface connective tissue were removed. The renal tissues with the size of 1 mm × 1 mm × 1 mm were fixed in glutaraldehyde solution with pH 7.2 to observe the ultrastructure of them. The renal tissues with the size of 5 mm × 5 mm × 3 mm were placed in 10% formalin phosphate buffer solution to observe the histopathological changes, apoptosis staining and immunohistochemistry. The remaining kidney tissues were quickly frozen in liquid nitrogen and then stored in a −80°C refrigerator for subsequent experiments to extract RNA and protein and prepare tissue homogenate.

### Measured parameters

#### Monitoring of body weight changes and kidney specific gravity in rats

The body weight of rats was measured every 3 days since the start of the experiment and measured once a day after the administration of cisplatin. The kidney tissues of rats were completely removed and weighed after being euthanized. Subsequently, the body weight difference and kidney specific gravity were calculated. (Body weight difference = body weight 72 h after cisplatin administration−body weight on the day of cisplatin administration. Kidney specific gravity = weight of kidney tissues/body weight on the day of cisplatin administration × 100%).

#### Measurement of BUN and Cre in serum

BUN and Cre levels in serum of rats were measured using the Urea Nitrogen assay kit and Creatinine assay kit (Nanjing Jiancheng Bioengineering Institute, Nanjing, China) according to the manufacturer’s instructions.

#### Histopathological examination

The kidney tissue samples were fixed in 4% paraformaldehyde (PFA) in saline for 24 h as previously described. Samples were then dehydrated with alcohols, cleared in xylene, embedded in paraffin, and cut into sections (4 μm thickness) using a Leica microtome (Leica, Weztlar, Germany). The prepared sections were placed on glass slides, deparaffinized, stained with hematoxylin-eosin (H&E), and examined under a light microscope (Nikon Corporation, Tokyo, Japan).

#### Transmission electron microscopy examination

The kidney tissue samples were placed in a 2.5% glutaraldehyde solution overnight, and then washed three times in 0.2 M phosphate buffer (pH 7.2) for 15 min each. Subsequently, the samples were immobilized in 1% osmium tetroxide for 100 min, and then washed in the same way. After dehydration with ethanol (50, 70, 90, and 100%) and 100% acetone, the renal tissues were immersed in anhydrous acetone and embedded solution (1:1, 1:2, 1:3) for 40 min, 2 h, and overnight, respectively. The slices were cut with an ultra-thin microtome (Leica, Weztlar, Germany) with a thickness of 50 ~ 60 nm and double stained with uranium acetate and examined under a H-7650 transmission electron microscope (Hitachi Corporation, Tokyo, Japan).

#### Measurement of oxidant status and antioxidant enzymes

Levels of MDA, hydrogen peroxide (H_2_O_2_), nitric oxide (NO), inducible nitric oxide synthase (iNOS), and activities of SOD, catalase (CAT), glutathione (GSH), total antioxidant capacity (T-AOC) in rat kidney tissue homogenates were determined using commercial kits (Nanjing Jiancheng Bioengineering Institute, Nanjing, China). The absorbance values were measured using a microplate spectrophotometer (Bio Tek Instruments, Vermont, USA) according to the manufacturer’s instructions.

#### Measurement of ATPase activities

Activities of Na^+^K^+^-ATPase, Ca^2+^-ATPase, and Ca^2+^Mg^2+^-ATPase in rat kidney tissue homogenates were measured using the ATPase assay kits (Nanjing Jiancheng Bioengineering Institute, Nanjing, China) according to the manufacturer’s instructions.

#### Apoptosis detection

Terminal deoxynucleotidyl transferase-mediated dUTP nick end labeling (TUNEL) assay was performed according to the instruction of *in situ* cell death detection kit (Roche Biomedical Laboratories Inc., Burlington, Germany). The protocol is as follows: the sections of the kidney tissue were fixed on 4% PFA in pH 7.2 PBS at room temperature for 20 min, and then washed 3 × 5 min in pH 7.2 PBS buffer. The sections were digested with pepsin at 37°C for 40 min and washed 3 × 10 min in PBS. Thirdly, the sections were incubated in enzyme reaction mix for 1 h at 37°C in a black wet box and then washed 3 × 10 min in PBS. Finally, the nuclei were stained with 4, 6-diamino-2-phenylindole (DAPI, Sigma-Aldrich Co., St. Louis, MO, USA) at room temperature for 30 min. The sections were cover-slipped with glycerol-PBS (3:1 v/v) and examined by Leica 4000 (Leica, Weztlar, Germany) at 488 and 350 nm. Normal cells and apoptotic cells can be distinguished according to the difference in cell color and nuclear morphology after staining. Normal cells are blue and apoptotic cells have fluorescence.

### qRT-PCR analysis

Quantitative reverse transcriptase polymerase chain reaction (qRT-PCR) was used to detect the mRNA abundance of NF-κB, TNF-α, IL-1β, IL-6, IL-10, B cell lymphoma-2 (Bcl-2)/Bcl-xl associated death promoter (Bad), Bcl-2, Cytochrome C (Cyt C), Caspase 9, Caspase 3, Caspase 8, and microtubule-associated protein 4 (MAP4). Using TRIzol reagent (Invitrogen, California, USA), total RNA was isolated from the frozen samples. Available concentration and purity of mRNA were retranscripted to cDNA strand using TransScript reverse transcriptase kits (Beijing TransGen Biotech Co. Ltd., Beijing, China) following the manufacturer’s instructions. All the primer sequences listed in Supplementary Table 1 were specifically validated and conformed to the normal distribution. SYBR Green master mix (Roche Biomedical Laboratories Inc., Burlington, Germany) and primers were used to amplify cDNA. The obtained amplification data were analyzed using glyceraldehyde-3-phosphate dehydrogenase (GAPDH) as internal parameter by the 2^-ΔΔCt^ method.

### Western blotting analysis

After the proteins were separated by electrophoreses, all samples were shifted to polyvinylidene difluoride membranes (Millipore Corporation, Massachusetts, USA). The membranes were incubated with the first Abs, NF-κB p65 (1:500), p-NF-κB p65 (1:500), TNF-α (1:500), Pro-IL-1β (1:1000), IL-6 (1:1000), IL-10 (1:500), Bad (1:750), Bcl-2 (1:500), Cyt C (1:1000), Cleaved-caspase 9 (1:500), Cleaved-caspase 3 (1:500), Cleaved-caspase 8 (1:1500), PI3K p110 (1:1000), AKT (1:500), p-AKT (1:500), MAP4 (1:500) (Wanlei Biotechnology Corporation, Shenyang, China), for 12~16 h at 4°C and then with the HRP conjugated anti-rabbit Ab (1:4000) (Wanlei Biotechnology Corporation, Shenyang, China) for 90 min at room temperature. Western blotting was monitored by using a chemiluminescence detection reagent (Meilun Biotechnology Corporation, Dalian, China) and visualized using Tanon 5200 Automatic Gel Imager (Tanon Science & Technology Corporation, Shanghai, China). For quantification, β-Tubulin (1:500) was used as the inner standards of tissue proteins.

### Immunohistochemistry

After the paraffin sections with a diameter of 1 cm and a thickness of 4 μm were deparaffinized, the rat kidney tissues were incubated with 3% H_2_O_2_ solution for 25 min. After rinsing the tissues with PBS, the tissues were blocked with 10% normal goat serum (Solarbio Science & Technology Co., Ltd., Beijing, China) at room temperature for 1 h, and then incubated with the primary antibody (MAP4) at 4°C overnight. Subsequently, an anti-rabbit IgG antibody (diluted 1:500) was used to react with the tissues at room temperature for 2 h. The tissues were then reacted with diaminobenzidine (DAB, Solarbio Science & Technology Co., Ltd., Beijing, China) for 10 min. Finally, images were obtained through microscope.

### Statistical analysis

All the data were analyzed using SPSS 22.0 statistical package (SPSS Inc., Chicago, IL). Comparisons among different experimental groups for statistical significance were performed using a one-way analysis of variance (ANOVA) and the Tukey-Kramer test. Data were reported as mean ± standard deviation (*M* ± SD). The data were plotted using GraphPad Prism 7 software. *P* > 0.05 was set to indicate no significant differences, *P <* 0.05 indicated significant differences, and *P <* 0.01 indicated extremely significant differences.

## Results

### Effects of troxerutin on weight change in rats

**Fig. 1:**
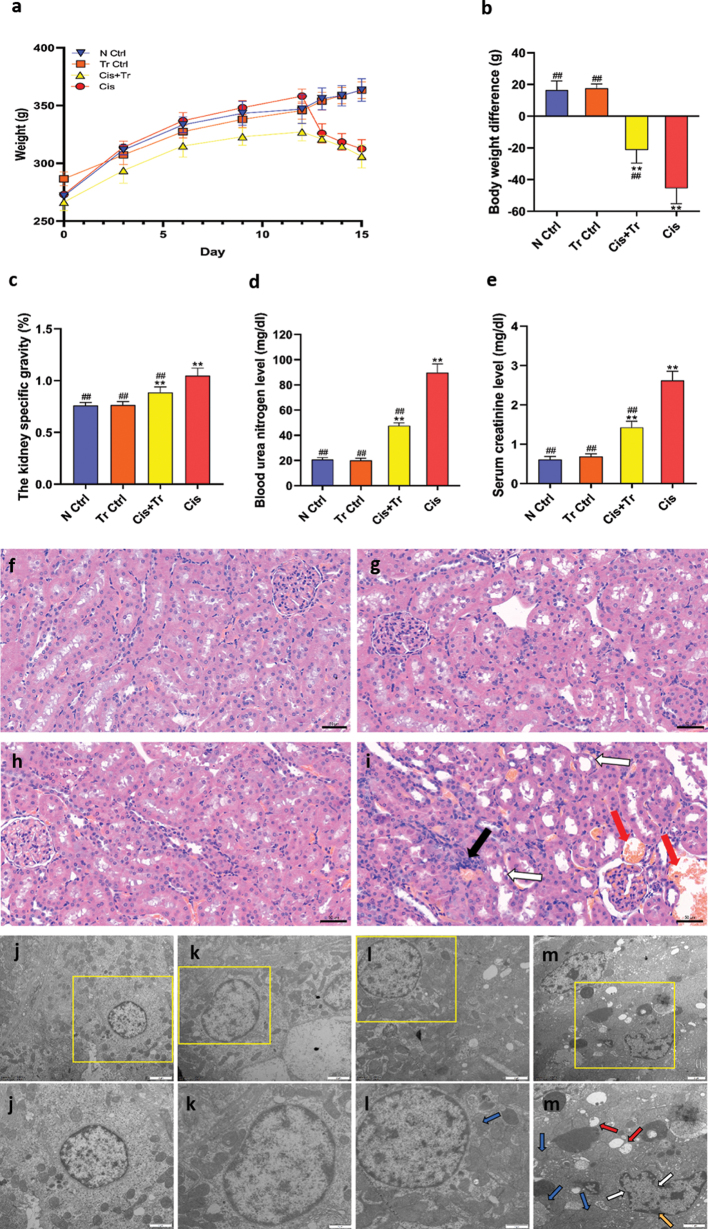
Troxerutin alleviated weight loss and renal dysfunction in rats. (a) Changes in body weight of rats. (b) Body weight difference. (c) The kidney specific gravity. (d) Blood urea nitrogen level. (e) Serum creatinine level. N Ctrl, normal control group; Tr Ctrl, troxerutin control group; Cis+Tr, cisplatin and troxerutin-treated group; Cis, cisplatin-treated group. Each bar represents the *M* ± SD of 10 animals. Statistical analysis by one-way ANOVA followed by Tukey-Kramer multiple comparisons. **P < 0.01 denotes comparison with the N Ctrl group. ^##^*P* < 0.01 denotes comparison with the Cis group. (f–i) H&E staining for the normal control group, troxerutin control group, cisplatin and troxerutin-treated group, and cisplatin-treated group, respectively. Scale bar = 50 μm. Black arrow, inflammatory cells infiltration; White arrow, vacuolar degeneration; Red arrow, erythrocytosis. (j–m) Transmission electron microscopy for ultrastructure assessment of kidney in the normal control group, troxerutin control group, cisplatin and troxerutin-treated group, and cisplatin-treated group, respectively. Scale bar = 2 μm. (j–m) Detail magnification from j–m. Scale bar = 1 μm. White arrow, the nuclear shrinkage; Red arrow, vacuolation; Yellow arrow, the chromatin edge aggregation; Blue arrow, mitochondrial cristae fracture.

The weight of rats in the N Ctrl group and Tr Ctrl group increased slightly, and the body weight difference was 16.5 ± 5.7 g and 17.6 ± 2.7 g, respectively. The rats in the Cis+Tr group and Cis group also gained weight before the administration of cisplatin. However, the weight dropped sharply after intraperitoneal injection of cisplatin. The body weight difference of the Cis+Tr group and Cis group was −21.2 ± 8.4 g and −45.5 ± 9.7 g, respectively (Fig. 1a, b).

### Troxerutin alleviated renal dysfunction in rats

To investigate cisplatin-induced renal function failure and the alleviating effect of troxerutin, the kidney specific gravity was calculated and is shown in Fig. 1c. The levels of BUN and Cre in serum were measured and are shown in Fig. 1d, e. All the data showed no significant difference between N Ctrl and Tr Ctrl groups (*P* > 0.05). The Cis+Tr group and Cis group exhibited a significant increase in kidney specific gravity (*P <* 0.01), serum BUN (*P <* 0.01), and Cre (*P <* 0.01) levels as compared to the N Ctrl group, suggesting kidney injury. On the contrary, troxerutin treatment significantly reduced the kidney specific gravity and the increased levels of BUN (*P <* 0.01) and Cre (*P <* 0.01) in serum with respect to the Cis group.

To investigate cisplatin-induced histopathological damage in kidney tissues and the alleviating effect of troxerutin, all groups were stained with H&E and are shown in Fig. 1f–i. The rat kidney tissue cells in the N Ctrl group and Tr Ctrl group were compactly arranged. The nucleus was of uniform size, showing a uniform light blue, and the cytoplasm was uniformly stained (Fig. 1f, g). Compared with the N Ctrl group, the kidney cells in the Cis group were disordered, and vacuolar degeneration occurred around the nucleus. There was an increase in erythrocytes between the renal tissues and an infiltration of inflammatory cells (Fig. 1i). Troxerutin treatment showed marked improvement with fewer erythrocytes, less vacuolar degeneration, and inflammatory cells infiltration compared with the Cis group (Fig. 1h).

The ultrastructure of the kidney from the N Ctrl and Tr Ctrl groups was normal in appearance, showing clear and complete cell morphology, and normal morphology of intracytoplasmic organelles (Fig. 1j–k). In the Cis group, there was acute injury accompanied with volume reduction of cells and cytoplasm vacuolation. The nucleus shrinked, and the nuclear membrane was not clear. Mitochondria were destroyed, and mitochondrial cristae were broken or even disappeared (Fig. 1m). Moreover, the damage was mild in the Cis+Tr group. The cell structure was clear and complete, the chromatin edge aggregation in the cytoplasm was not obvious, the cytoplasm was slightly vacuolated, and some mitochondrial cristae were broken (Fig. 1l).

### Troxerutin alleviated renal oxidative stress

**Fig. 2:**
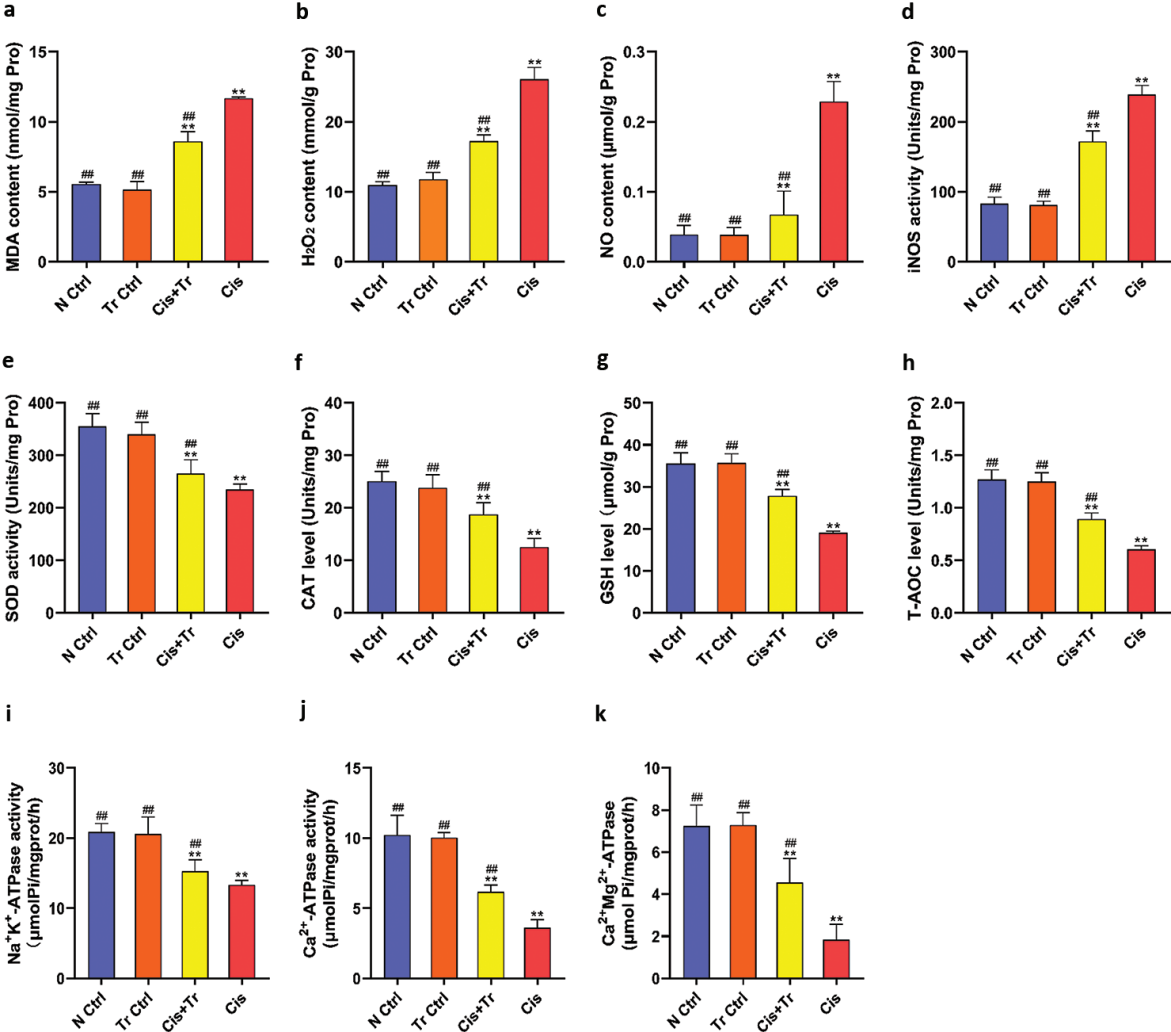
Troxerutin alleviated renal oxidative stress and improved ATPase activities. (a) MDA content. (b) H_2_O_2_ content. (c) NO content. (d) iNOS activity. (e) SOD activity. (f) CAT level. (g) GSH level. (h) T-AOC level. (i) Na^+^K^+^-ATPase activity. (j) Ca^2+^-ATPase activity. (k) Ca^2+^Mg^2+^-ATPase activity. N Ctrl, normal control group; Tr Ctrl, troxerutin control group; Cis+Tr, cisplatin and troxerutin-treated group; Cis, cisplatin-treated group. Each bar represents the *M* ± SD of 10 animals. Statistical analysis by one-way ANOVA followed by Tukey-Kramer multiple comparisons. **P < 0.01 denotes comparison with the N Ctrl group. ^##^*P* < 0.01 denotes comparison with the Cis group.

To investigate cisplatin-induced oxidative stress and the rescue role of troxerutin, oxidant status (MDA, H_2_O_2_, NO, and iNOS) and the activities of relative antioxidant enzymes (SOD, CAT, GSH, and T-AOC) in all groups of kidneys were measured and are shown in Fig. 2a–h. All data showed no significant differences between N Ctrl and Tr Ctrl groups (*P* > 0.05). The activities of SOD, CAT, GSH, and T-AOC in the Cis group were significantly decreased (*P <* 0.01), while the levels of MDA, H_2_O_2_, NO, and iNOS were significantly increased (*P <* 0.01) compared to the N Ctrl group. Compared with the Cis group, the activities of SOD, CAT, GSH, and T-AOC in the Cis+Tr group were significantly increased (*P <* 0.01), while the levels of MDA, H_2_O_2_, NO, and iNOS were significantly decreased (*P <* 0.01).

### Troxerutin improved renal ATPase activities

The activities of Na^+^K^+^-ATPase,Ca^2+^-ATPase, and Ca^2+^Mg^2+^-ATPase were measured and are shown in Fig. 2i–k. All data showed no significant difference between the N Ctrl group and the Tr Ctrl group (*P* > 0.05). ATPase activities in the Cis group were significantly decreased compared to the N Ctrl group (*P <* 0.01), while the Cis+Tr group had an increased effect on ATPase activities compared to the Cis group (*P <* 0.01).

### Troxerutin alleviated renal inflammation

**Fig. 3:**
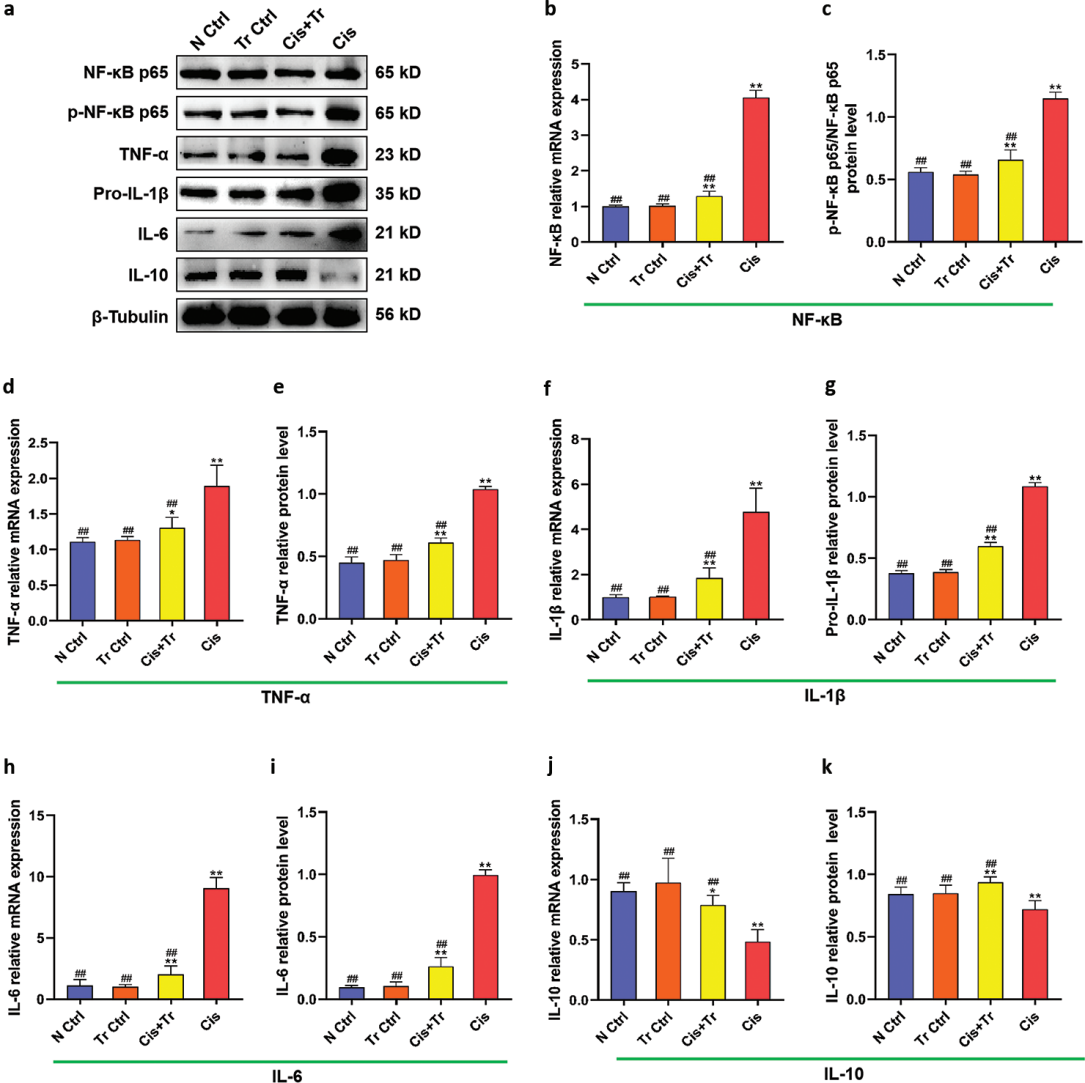
Troxerutin alleviated renal inflammation. (a) Western blotting of NF-κB p65, p-NF-κB p65, TNF-α, Pro-IL-1β, IL-6, and IL-10. (b) NF-κB relative mRNA expression. (c) p-NF-κB p65/NF-κB p65 protein level. (d) TNF-α relative mRNA expression. (e) TNF-α relative protein level. (f) IL-1β relative mRNA expression. (g) Pro-IL-1β relative protein level. (h) IL-6 relative mRNA expression. (i) IL-6 relative protein level. (j) IL-10 relative mRNA expression. (k) IL-10 relative protein level. N Ctrl, normal control group; Tr Ctrl, troxerutin control group; Cis+Tr, cisplatin and troxerutin-treated group; Cis, cisplatin-treated group. Each bar represents the *M* ± SD of 10 animals. Statistical analysis by one-way ANOVA followed by Tukey-Kramer multiple comparisons. *P < 0.05 and **P < 0.01 denote comparison with the N Ctrl group. ^##^*P* < 0.01 denotes comparison with the Cis group.

To confirm the effect of troxerutin on cisplatin-induced inflammation in kidney, genes of NF-κB, TNF-α, and IL-1β/6/10 and proteins of NF-κB p65, p-NF-κB p65, TNF-α, Pro-IL-1β, and IL-6/10 were detected by qRT-PCR and western blotting in each group (Fig. 3). The results of the N Ctrl group and the Tr Ctrl group were similar (*P* > 0.05). Compared with the N Ctrl group, the mRNA levels of NF-κB, TNF-α, and IL-1β/6 and protein levels of p-NF-κB p65/NF-κB p65, TNF-α, Pro-IL-1β, and IL-6 in the Cis group were significantly upregulated (*P <* 0.01), while troxerutin treatment downregulated these levels (*P <* 0.01). On the contrary, the mRNA and protein levels of IL-10 showed a significant suppression in the Cis group (*P <* 0.01), while the levels of IL-10 were upregulated in the Cis+Tr group (*P <* 0.01).

### Troxerutin alleviated renal cell apoptosis

**Fig. 4:**
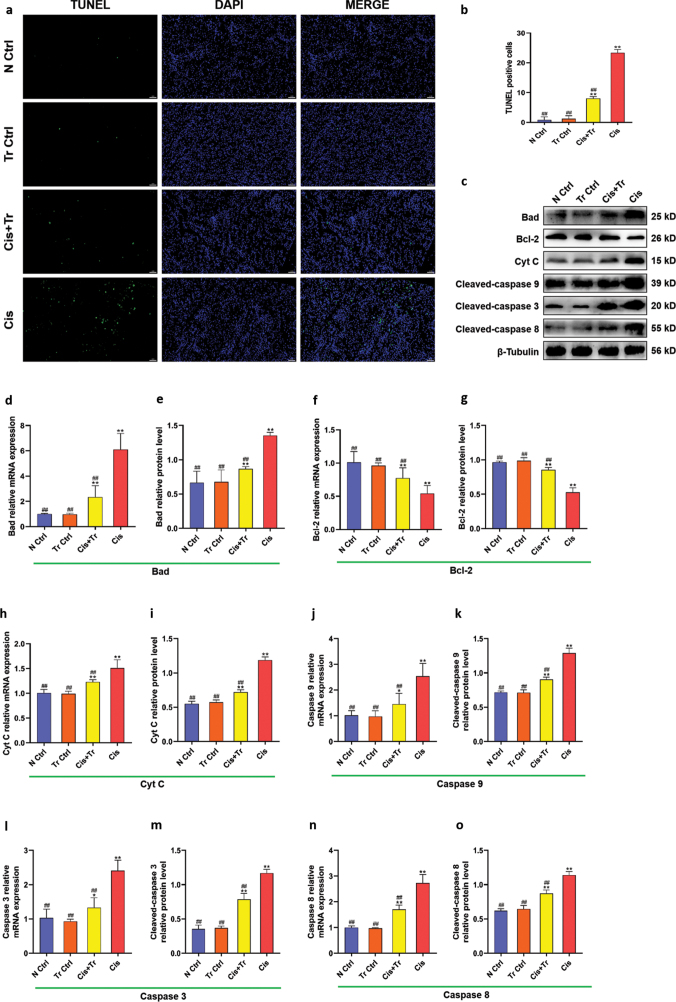
Troxerutin alleviated renal cell apoptosis. (a) TUNEL assay of renal cell apoptosis. Scale bar = 50 μm. (b) TUNEL positive cells. (c) Western blotting of Bad, Bcl-2, Cyt C, Cleaved-caspase 9, Cleaved-caspase 3, and Cleaved-caspase 8. (d) Bad relative mRNA expression. (e) Bad relative protein level. (f) Bcl-2 relative mRNA expression. (g) Bcl-2 relative protein level. (h) Cyt C relative mRNA expression. (i) Cyt C relative protein level. (j) Caspase 9 relative mRNA expression. (k) Cleaved-caspase 9 relative protein level. (l) Caspase 3 relative mRNA expression. (m) Cleaved-caspase 3 relative protein level. (n) Caspase 8 relative mRNA expression. (o) Cleaved-caspase 8 relative protein level. N Ctrl, normal control group; Tr Ctrl, troxerutin control group; Cis+Tr, cisplatin and troxerutin-treated group; Cis, cisplatin-treated group. Each bar represents the *M* ± SD of 10 animals. Statistical analysis by one-way ANOVA followed by Tukey-Kramer multiple comparisons. *P < 0.05 and **P < 0.01 denote comparison with the N Ctrl group. ^##^*P* < 0.01 denotes comparison with the Cis group.

TUNEL assay was performed to investigate the protective effect of troxerutin on cisplatin-induced renal cell apoptosis. The results of staining showed that only a small amount of green fluorescence and fewer apoptotic cells were found in the N Ctrl group, Tr Ctrl group, and Cis+Tr group. There were more green fluorescence and more apoptotic cells in the Cis group (Fig. 4a–b).

Cytochrome C (Cyt C) binds with apoptotic enzyme activator-1 (Apaf-1) to form Apaf-1/Cyt C apoptotic complex, which can activate Caspase 9, and then activate Caspase 3, promoting cell apoptosis ultimately (18). In addition, death receptors TNFR1, TNFR2 (tumor necrosis factor receptor), and Fas are bounded and activate Caspase 8, and then activate Caspase 3, leading to cell apoptosis. Genes of Bad, Bcl-2, Cyt C, Caspase 9, Caspase 3, and Caspase 8 were detected by qRT-PCR, and proteins of Bad, Bcl-2, Cyt C, Cleaved-caspase 9, Cleaved-caspase 3, and Cleaved-caspase 8 were detected by western blotting, as shown in Fig. 4c–o. The results of the N Ctrl group and the Tr Ctrl group were almost identical (*P* > 0.05). Compared with the N Ctrl group, the mRNA levels of Bad, Cyt C, Caspase 9, Caspase 3, and Caspase 8 and protein levels of Bad, Cyt C, Cleaved-caspase 9, Cleaved-caspase 3, and Cleaved-caspase 8 in the Cis group were significantly upregulated (*P <* 0.01). However, these levels in the Cis+Tr group were downregulated compared to the Cis group (*P <* 0.01). The mRNA and protein levels of Bcl-2 were downregulated in the Cis group (*P <* 0.01), while troxerutin treatment could upregulate the levels of Bcl-2 (*P <* 0.01).

### Troxerutin regulated PI3K/AKT pathway via enhancing MAP4 expression

**Fig. 5:**
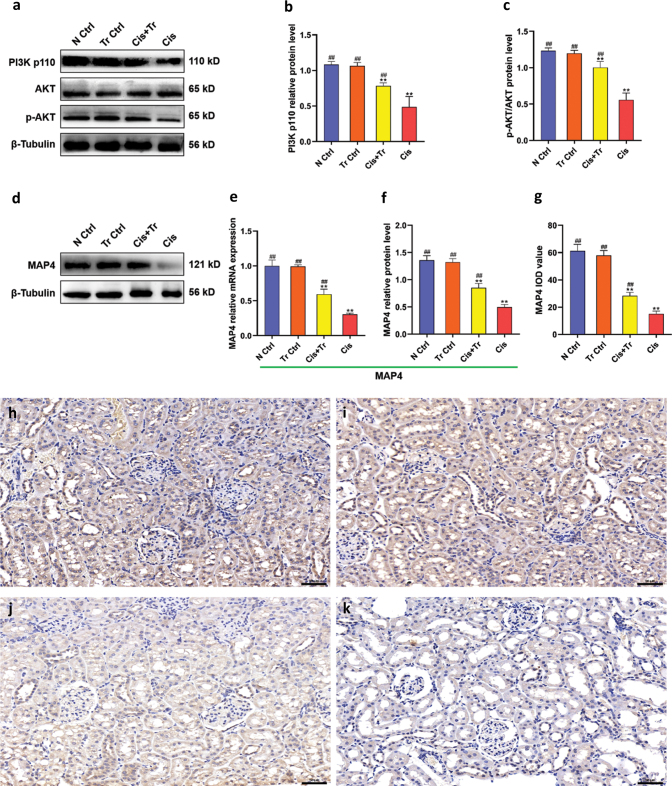
Troxerutin regulated PI3K/AKT pathway via enhancing MAP4 expression. (a) Western blotting of PI3K p110, AKT, and p-AKT. (b) PI3K p110 relative protein level. (c) p-AKT/AKT protein level. (d) Western blotting of MAP4. (e) MAP4 relative mRNA expression. (f) MAP4 relative protein level. (g) MAP4 IOD value. (h–k) Immunohistochemistry analysis showing the distribution and abundance of MAP4 in the normal control group, troxerutin control group, cisplatin and troxerutin-treated group, and cisplatin-treated group, respectively. Scale bar = 50 μm. N Ctrl, normal control group; Tr Ctrl, troxerutin control group; Cis+Tr, cisplatin and troxerutin-treated group; Cis, cisplatin-treated group. Each bar represents the *M* ± SD of 10 animals. Statistical analysis by one-way ANOVA followed by Tukey-Kramer multiple comparisons. **P < 0.01 denotes comparison with the N Ctrl group. ^##^*P* < 0.01 denotes comparison with the Cis group.

The Phosphoinositide 3 kinase/Protein kinase B (PI3K/AKT) signaling pathway plays a regulatory role in substance metabolism, cell cycle, and apoptosis, which has important research value. To evaluate the effects of troxerutin on the PI3K/AKT signaling pathway in kidney, gene of MAP4 and proteins of PI3K p110, AKT, p-AKT, MAP4 in various groups were determined and are shown in Fig. 5. Compared with the N Ctrl group, the protein levels of PI3K p110, p-AKT/AKT in the Cis group were suppressed (*P <* 0.01), while troxerutin increased them (*P <* 0.01) (Fig. 5a–c). The transcription level and protein level of MAP4 were significantly reduced in the Cis group (*P <* 0.01); however, these levels in the Cis+Tr group were increased compared to the Cis group (*P <* 0.01) (Fig. 5d–f). Moreover, the immunohistochemical results showed the expression and localization of MAP4 in each group. The positive expression of MAP4 was found in the renal tubules. The chromogenic intensity of the Cis group was significantly lower than that of the N Ctrl group, suggesting that the expression of MAP4 was decreased in the cisplatin-induced kidney injury model. However, the chromogenic intensity of the Cis+Tr group was significantly increased compared with the Cis group (Fig. 5g–k).

## Discussion

Cisplatin is often used to treat various solid organ cancers, but it can also cause kidney injury by stimulating the body to produce oxidative stress, inflammation, apoptosis, autophagy, and vascular damage (17, 19). There is no specific drug that can prevent and treat the kidney injury of cisplatin currently. Research on its protective measures has always been a research hotspot in recent years. At present, hydration and diuretic measures are mainly taken clinically for protection. According to the different mechanisms of cisplatin-induced renal injury, multiple emerging protective drugs have also been proposed. Excretion of cisplatin in renal tissues requires the involvement of organic cationic transporter (OCT) 2, copper transporter (Ctr) 1, and multidrug and toxin extrusion (MATE) 1. Increasing the activity of OCT 2, Ctr 1 and/or decreasing the activity of MATE 1 can enhance the nephrotoxicity of cisplatin (20). Katsuda et al. (21) found that cimetidine, as an OCT inhibitor, can significantly inhibit the nephrotoxicity, but had no significant effect on the anti-tumor activity of cisplatin both *in vivo* and *in vitro*. In addition, it has been reported that certain nutrients (22, 23) and chemical drugs (24) can prevent cisplatin-induced nephrotoxicity. In recent years, plant-derived preparations have become a hot topic as protective agents, including Chinese medicine-derived preparations such as esperidin (25), and food-derived preparations such as tea polyphenols (26). Troxerutin, also a food-derived preparation, has been used as a potential protective agent against organ injury in recent years. Salama et al. (8) found that troxerutin may improve gentamicin-induced renal injury in rats by regulating p38 MAPK signaling transduction as well as antioxidant, anti-inflammatory, and anti-apoptotic activities. Shan et al. (11) found that troxerutin can regulate the inflammatory lesions via CXCR4-TXNIP/NLRP3 inflammasome in the kidney of mice induced by BDE-47. Jamali-Raeufy et al. (27) found that troxerutin can inhibit lipopolysaccharide (LPS)-induced acute neuroinflammation, oxidative stress, apoptosis, and subsequently memory impairments by targeting SIRT1/SIRT3 signaling pathway. Sui et al. (13) found that troxerutin and cerebroprotein can alleviate cerebral I/R injury by downregulating caspase-1/3/8.

In our present study, we found that serum levels of BUN and Cre were higher in cisplatin-treated rats compared to the normal control rats, but troxerutin-treated rats significantly reduced the increase. Furthermore, the histopathological damage of renal tissue in cisplatin-treated rats included interstitial inflammatory cell infiltration, vacuolar degeneration, and increased red blood cells. The ultrastructure of them was mainly changed by cytoplasmic vacuolization, pyknosis of the nucleus, and mitochondrial cristae breakage and even disappearance. The rats in the Cis+Tr group showed normal histological morphology and ultrastructure, which confirmed that troxerutin can alleviate cisplatin-induced kidney injury in rats.

Oxidative stress is associated with cisplatin-induced kidney injury. Any factor that enhances oxidation and/or reduces antioxidant capacity may increase reactive oxygen species (ROS), which in turn puts the body in a state of oxidative stress (28). We found that the contents of MDA, H_2_O_2_, and NO and the activity of iNOS in the kidney tissues of rats treated with cisplatin increased, while the activities of SOD, CAT, GSH, and T-AOC decreased compared to the N Ctrl group. However, troxerutin can reverse these changes. The anti-oxidative effects of troxerutin were established in other nephrotoxic models, such as D-gal-induced kidney injury (10), gentamycin-induced acute kidney injury (8), unilateral ureteral obstruction-induced nephropathy (29).

In addition, cisplatin can reduce the activity of Na^+^K^+^-ATPase and Ca^2+^Mg^2+^-ATPase and inhibit the function of mitochondrial complex I, II, III, and IV, thereby reducing the level of intracellular ATP, which may cause the disorder of energy synthesis and the destruction of antioxidant defense system (30). Our observations also supported the results of Tadini-Buoninsegni et al. (31), who have shown that cisplatin inhibited Na^+^K^+^-ATPase activity, and we also found that Ca^2+^-ATPase and Ca^2+^Mg^2+^-ATPase activities were inhibited. On the contrary, troxerutin had a significant protective effects on ATPase activities in rat renal tissue.

Inflammation is another pathogenic mechanism of cisplatin-induced renal injury. Cisplatin increases the serum concentration of TNF-α, a pleiotropic cytokine with endocrine, paracrine, and autocrine pro-inflammatory consequences, which can trigger systemic inflammatory networks (32). Furthermore, tumor necrosis factor (TNF) has been shown to activate NF-κB, and many biological effects of TNF can be mediated by NF-κB (33). In our present study, we found that the expressions of NF-κB phosphorylation, TNF-α, and the downstream pro-inflammatory factors IL-1β, IL-6 were higher, and the expression of anti-inflammatory factor IL-10 was lower in the renal tissue of rats treated with cisplatin compared to normal control rats. Troxerutin not only decreased the transcription and translation levels of NF-κB, TNF-α, IL-1β, and IL-6, but also increased the level of IL-10, which confirmed the anti-inflammatory activity of troxerutin. This ability was demonstrated in other previous trials. Wang et al. (34) found that troxerutin can improve dextran sulfate sodium-induced ulcerative colitis in mice by reducing the infiltration of inflammatory cells and decreasing the expression of inflammation-related proteins and proinflammatory cytokines in the colon tissue. Badalzadeh et al. (35) found that both pretreatment with troxerutin and postconditioning can reduce the levels of TNF-α and IL-1β, alleviating myocardial I/R injury in rats. Jafari-Khataylou et al. (36) found that troxerutin was an effective anti-inflammatory agent for the treatment of LPS-induced sepsis, which can reduce inflammatory parameters, improve antioxidant activity, and improve survival rate of mice.

Studies have shown that cisplatin-induced apoptosis of renal tubular epithelial cells is mainly achieved through the endogenous pathway mediated by mitochondria and the exogenous pathway mediated by death receptors (37). We found that the pro-apoptotic protein Bad was upregulated, the anti-apoptotic protein Bcl-2 was downregulated, and caspase was activated in cisplatin-induced kidney injury. In addition to priming inflammatory response, TNF-α can stimulate the expression of Caspase 8 and initiate exogenous cell apoptosis. Troxerutin mitigated nephrotoxicity of cisplatin by attenuating the endogenous and exogenous cell apoptosis in cisplatin-injected rats. Moreover, the TUNEL test results showed that there were more apoptotic cells in the Cis group, and the number of apoptotic cells in the Cis+Tr group decreased, which once again confirmed the anti-apoptotic ability of troxerutin.

Our data also demonstrated for the first time that troxerutin might attenuate cisplatin-induced renal cell apoptosis in rats through activating the PI3K/AKT signaling pathway by enhancing MAP4 expression. The PI3K/AKT pathway is one of the most potent intracellular mechanisms to promote cell survival (38). PI3K is the only downstream effector of growth receptors that can continue to inhibit apoptosis after serum withdrawal. AKT regulates a series of downstream targets genes of apoptosis, such as Bad and Caspase 9 which can be inactivated after AKT phosphorylation (39). We found that cisplatin can reduce the protein expression levels of PI3K p110 and p-AKT/AKT, and troxerutin can activate the PI3K/AKT pathway to alleviate rat renal injury. These observations are similar to the results of Li et al. (40), who have shown that corin can attenuate cell apoptosis through activation of PI3K/AKT pathway, thereby protecting cardiomyocytes from H_2_O_2_-induced damage. Shu et al. (41) found that troxerutin pretreatment can significantly improve myocardial I/R injury, and the protective effect was mediated by the PI3K/AKT pathway. Troxerutin upregulated the protein level of p-AKT in a dose-dependent and time-dependent manner. Microtubules are a major component of eukaryotic cytoskeleton, and microtubule-associated proteins (MAPs) are important components for binding and stabilizing microtubules (42). According to a recent study, as a member of the MAPs family and a binding partner of the p110α catalytic subunit of PI3K (43), MAP4 can provide a platform for the distribution of PI3K along microtubules and its binding to the corresponding receptors. Thapa et al. (43) also found that the interaction between MAP4 and PI3K is a necessary condition for the production of phosphatidylinositol 3,4,5-triphosphate (PIP3). They applied mutant cells that continuously expressed PI3K activity but lacked MAP4 to conduct experiments, and the results showed that MAP4 is ubiquitous for PI3K signaling pathway. Our observations demonstrated that the transcription and translation levels of MAP4 decreased in kidney tissue of rats in the Cis+Tr group, while troxerutin increased the expression of MAP4 and relieved tubulin damage compared to the Cis group, which was in line with the above point of view.

## Conclusions

In conclusion, these results revealed the protective effect of troxerutin against cisplatin-induced kidney injury, and the possible underlying mechanism might be attributed to the regulation of PI3K/AKT pathway via enhancing MAP4 expression to attenuate cellular apoptosis, alleviating oxidative stress and inflammatory response. Further *in vivo* and *in vitro* studies are warranted to evaluate the other mechanisms of troxerutin effects in cisplatin-induced kidney injury model. In addition, we can continue to explore the relationship between troxerutin and different organs as well as signaling pathways, which has certain research value.

## Supplementary Material

Troxerutin alleviates kidney injury in rats via PI3K/AKT pathway by enhancing MAP4 expression

Troxerutin alleviates kidney injury in rats via PI3K/AKT pathway by enhancing MAP4 expression
