# Supplementary material for: Troxerutin alleviates kidney injury in rats via PI3K/AKT pathway by enhancing MAP4 expression
Source: Food Nutr Res. 2022 May 24;66:10.29219/fnr.v66.8469. doi: 10.29219/fnr.v66.8469 (PMC9252313; doi:10.29219/fnr.v66.8469)
Supplement: Troxerutin alleviates kidney injury in rats via PI3K/AKT pathway by enhancing MAP4 expression [file FNR-66-8469-s001.pdf]

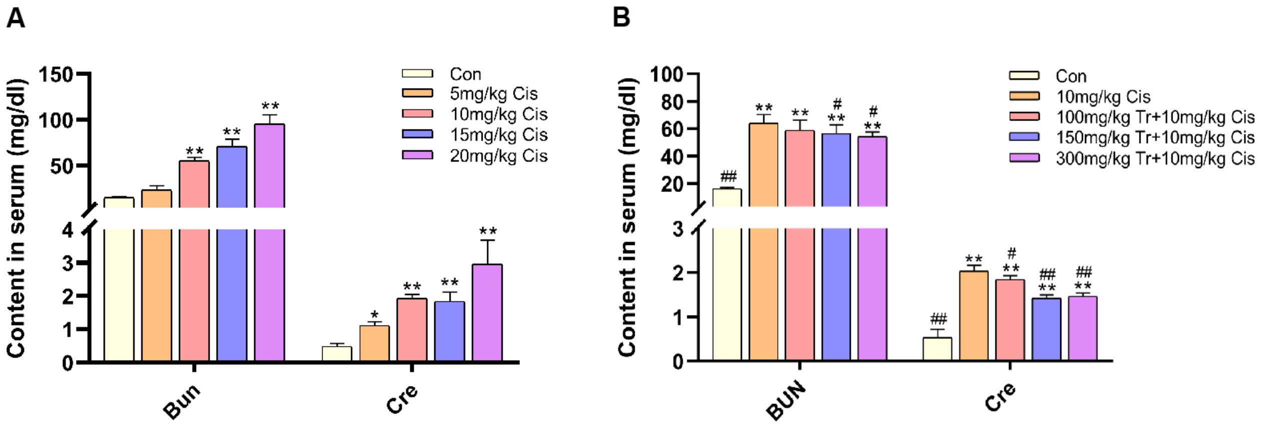

Fig.S1 Dosage determination of troxerutin and cisplatin. (A) Content of BUN and Cre in serum after administration of 5, 10, 15, 20mg/kg of cisplatin, respectively. (B) Content of BUN and Cre in serum after administration of 10mg/kg of cisplatin, 100mg/kg of troxerutin and 10mg/kg of cisplatin, 150mg/kg of troxerutin and 10mg/kg of cisplatin, 300mg/kg of troxerutin and 10mg/kg of cisplatin, respectively. Each bar represents the M±SD of 5 animals. Statistical analysis by one-way ANOVA followed by Tukey-Kramer multiple comparisons. $^{*}p<0.05$, $^{**}p<0.01$ denote comparison with the Con group. $^{\#}p<0.05$, $^{\#\#}p<0.01$ denote comparison with the 10mg/kg Cis group.
