# Supplementary material for: Troxerutin alleviates kidney injury in rats via PI3K/AKT pathway by enhancing MAP4 expression
Source: Food Nutr Res. 2022 May 24;66:10.29219/fnr.v66.8469. doi: 10.29219/fnr.v66.8469 (PMC9252313; doi:10.29219/fnr.v66.8469)
Supplement: Troxerutin alleviates kidney injury in rats via PI3K/AKT pathway by enhancing MAP4 expression [file FNR-66-8469-s002.pdf]

Tab.S1 Primer sequences for qRT-PCR.

Gene	Sequence (5' - 3')
TNF-α	F: TCTCAAGCCTCAAGTAACAAGC
	R: ATGAGGTAAAGCCCGTCAGC
IL-1β	F: ACAGATGAAGTGCTCCTTCCA
	R: GAATTCAAATGCTCCTTGATTTCT
IL-6	F: CCACCCACAACAGACCAGTA
	R: ACTCCAGAAGACCAGAGCAG
IL-10	F: ACTGCTATGTTGCCTGCTCTTACT
	R: GAATTCAAATGCTCCTTGATTTCT
Bad	F: CGGGACAGGCAGCCAATAAC
	R: ATTGCACGCACCGGAAGGAAC
Bcl-2	F: TGGAGAGCGTCAACAGGGAGATG
	R: GGTGTGCAGATGCCGGTTCAG
Cyt C	F: TTTGTTCAAAAGTGTGCCCAGTGC
	R: AGCAGCCTGGCCTGTCTTCC
Caspase 9	F: TCCTTGTGTCCTACTCCACCTTCC
	R: TCTTCCGAGCGAGCCCACTG
Caspase 3	F: ACGAACGGACCTGTGGACCTG
	R: GTTTCGGCTTTCCAGTCAGACTCC
Caspase 8	F: GTTCACACCATTCTCCTGCC
	R: GAAACCCCGTCTCTACTAAA
MAP4	F: TGCGTTTCCCCAGAGGTTAC
	R: TTTGTCCTGCCATCATTTCCA
GAPDH	F: TCTGCTCCTCCCTGTTCTAGAGACA
	R: TCAGGTGAGCCCCAGCCTTCT
